# Fifty years of the integrated control concept: moving the model and implementation forward in Arizona††

**DOI:** 10.1002/ps.1861

**Published:** 2009-10-27

**Authors:** Steven E Naranjo, Peter C Ellsworth

**Affiliations:** a USDA-ARS, Arid-Land Agricultural Research Center21881 N. Cardon Lane, Maricopa, AZ 85138, USA; b Department of Entomology, University of Arizona, Maricopa Agricultural Center37860 W. Smith-Enke Road, Maricopa, AZ 85138, USA

**Keywords:** *Bemisia tabaci*, decision aids, conservation biological control, selective insecticides, bioresidual, ecosystem services

## Abstract

Fifty years ago, Stern, Smith, van den Bosch and Hagen outlined a simple but sophisticated idea of pest control predicated on the complementary action of chemical and biological control. This integrated control concept has since been a driving force and conceptual foundation for all integrated pest management (IPM) programs. The four basic elements include thresholds for determining the need for control, sampling to determine critical densities, understanding and conserving the biological control capacity in the system and the use of selective insecticides or selective application methods, when needed, to augment biological control. Here we detail the development, evolution, validation and implementation of an integrated control (IC) program for whitefly, *Bemisia tabaci* (Genn.), in the Arizona cotton system that provides a rare example of the vision of Stern and his colleagues. Economic thresholds derived from research-based economic injury levels were developed and integrated with rapid and accurate sampling plans into validated decision tools widely adopted by consultants and growers. Extensive research that measured the interplay among pest population dynamics, biological control by indigenous natural enemies and selective insecticides using community ordination methods, predator:prey ratios, predator exclusion and demography validated the critical complementary roles played by chemical and biological control. The term ‘bioresidual’ was coined to describe the extended environmental resistance from biological control and other forces possible when selective insecticides are deployed. The tangible benefits have been a 70% reduction in foliar insecticides, a >$200 million saving in control costs and yield, along with enhanced utilization of ecosystem services over the last 14 years. Published in 2009 by John Wiley & Sons, Ltd.

## INTRODUCTION

Fifty years ago, four entomologists from California articulated a concept of pest management^1^ that has since become not only a seminal scientific contribution but also a driving force and conceptual foundation for all modern integrated pest management (IPM) programs. The integrated control concept (ICC), as it was coined,^1^ is ‘Applied pest control that combines and integrates biological and chemical control. Chemical control is used as necessary and in a manner that is least disruptive to biological control’.

This embodies what at first appears to be a simple set of requirements, but upon further reflection points to multiple key components that must be assembled strategically to produce the desired outcome. The four essential elements include thresholds for determining the need to control pest populations, sampling plans for measuring critical densities, an understanding of the impact of biological control on the pest and finally knowledge of selective insecticides or methods of deployment that complement rather than disrupt this biological control. The ICC was based around Stern, Smith, van den Bosch and Hagen's experiences in field crops in California, including alfalfa, cotton and safflower, but the incredible insights into the basic ecology that underpin control systems were then, and still are, boundless.

Integrated control (IC) has been an elusive goal for most modern-day integrated pest management (IPM) programs. There has always been an uneasy tension between biological control specialists and IPM scientists having to develop and deploy usable programs for client growers and pest management practitioners. Stern *et al.*^1^ had a depth of vision that still today we are trying to emulate through validated IC programs. Few examples exist in the literature (e.g. references 2 to 5), and even then specific IC elements may be missing or not fully addressed and validated. Still fewer examples of IC can be found in general grower practice. At some level, this represents neglect on the part of the scientific community which has not invested in the research and documentation needed to identify and promote IC. At another level, this underscores the difficulty of conducting systems-level research and development including elements of implementation and validation.

The implementation and validation of IC requires several postulates:

1. The biological control agents must be present and abundant in the untreated system.
2. The biological control agents must be able to survive, at some level, the application of selective controls in the system of interest.
3. Some functional assessment of conservation biological control must be conducted.
4. An interval of pest suppression (or degree of control) in excess of the chemical residual must be possible when a selective control is implemented.
5. The interval (or degree) of suppression is lost, or at least significantly reduced, when either control agent (conserved agent or selective control) is removed from the system.

Additionally, IC is predicated on efficient and validated sampling plans used in deployment of research-based economic injury levels (EILs) and economic thresholds (ETs).

In this review we will attempt to show how these multiple elements have been crafted, delivered and incorporated into an IC program for the whitefly, *Bemisia tabaci* (Genn.), in the Arizona cotton system. We will discuss development of decision tools based on economic thresholds and simple, but accurate, sampling plans. We will show that a rich beneficial arthropod fauna creates a flexible and resilient food web where 3–5 insect predator species dominate and remain viable control agents of *B. tabaci* even with well-timed applications of selective insecticides. Will we discuss multiple functional assessments of conserved biological control agents over many years will be discussed, including detailed life tables, analyses of marginal mortality rates, irreplaceable mortality and predator:prey ratios. We will show how these studies confirm the key role of predation in the cotton system, along with other natural abiotic sources of mortality such as weather. Furthermore, we will demonstrate the dynamic trade-off between the two key mortality factors, insecticides and predation, and show how irreplaceable mortality due to predation increases over time when selective, but not conventional broad-spectrum, chemistry is deployed. We demonstrate that the period of suppression can be measured in days for conventional chemistry versus weeks for selective chemistry, and the reintroduce concept of ‘bioresidual’, which was imparted to growers to communicate the extended suppressive interval made possible by selective insecticides and a functional IC program. The interdependence of chemical and biological control via predator exclusion studies will also be shown. Finally, we discuss the impact of our IC program as part of an overall IPM strategy and provide some perspective on the future.

## THE SYSTEM

Cotton is cultivated in about 80 countries, with a total production of ≈23.6 billion kg of lint in 2008,^6^ is plagued by dozens of arthropod pests,^7^,^8^ and has historically been exposed to large volumes of insecticides.^9^,^10^ In Arizona, cotton has been an important agricultural commodity for many decades, with recent production exceeding 260 000 ha in the early 1980s. There are three key pests of cotton in Arizona and other low desert production areas in California and Mexico, the pink bollworm (*Pectinophora gossypiella* Saunders), Lygus bugs (primarily *Lygus hesperus* Knight) and the sweetpotato whitefly (*B. tabaci*), all of which can severely impact upon yield and lint quality in any given year. Several other pests can be occasionally problematic, depending on year, region and ongoing pest control tactics that might disrupt natural control processes. Some of the foundational elements of IC penetrated Arizona during the 1950s and continued into the 1970s with the implementation of supervised control.^11^ Stern *et al.*^1^ defined supervised control as ‘Control of insects … supervised by qualified entomologists and based on conclusions reached from periodically measured population densities of pests and beneficial species’. Supervised control was in effect a precursor to IC and introduced some of the same elements, including sampling and thresholds, but without the attendant theoretical basis found in the ICC. In the program discussed by Carruth and Moore,^11^ a cooperative, grower-sponsored, weekly scouting program was implemented on about 5000 ha of cotton in southeastern Arizona over a 3 year period in response to heavy infestations of *P. gossypiella* in prior years that resulted in six applications of insecticides per field, precipitated secondary outbreaks of cotton leaf perforator and caused heavy mortality of honey bees. In the end, the scouting program cost the growers $4.08 ha^−1^ and reduced insecticide use by 71–84%, testifying to the potential power of prescriptive pest control outlined by Stern *et al.*^1^ as part of the ICC.

### The pest

*Bemisia tabaci* was first described as an agricultural pest in Greece 120 years ago^12^ and has since become one of the most devastating insect pests in the world, affecting multiple agronomic and horticultural crops. It is cosmopolitan in distribution, with outdoor populations limited to tropical and subtropical regions of the world, but through commercial trade it has established itself as a pest of many glasshouse production systems throughout temperate areas of Asia, Europe and North America. Unusual for aleyrodids, *B. tabaci* is highly polyphagous, with a historical host range of over 500 plants^13^,^14^ that continues to expand (e.g. reference 15). The pest causes direct feeding damage through the removal of phloem sap, vectors over 110 plant viruses,^16^ causes a number of feeding-induced plant disorders and can contaminate cotton lint and degrade produce through the deposition of honeydew.^17^,^18^ *Bemisia tabaci* has been known from Arizona since the 1920s^19^ but was associated with only sporadic pest issues until the early 1990s.^20^,^21^ This renewed pest status was due to the introduction of a new biotype (biotype B) that first invaded Florida in the mid-1980s, subsequently spread to the west coast by 1991 and rapidly displaced the indigenous biotype.^22^,^23^ The new B-biotype of *B. tabaci* shared many of the same general biological features of the indigenous biotype but in a more virulent form, including a broader host plant range, greater reproductive potential, enhanced mobility and a greater propensity to develop resistance to insecticides, among other qualities.^22^ The invasion of this new biotype wreaked havoc on existing pest management practices in Arizona and elsewhere and initially led to some of the costliest impacts on yield, lint quality and control measures ever recorded in the state.^24^

Like the 1954 episode with spotted alfalfa aphid threatening the California alfalfa industry^1^ (see Section 4.2), the Arizona whitefly outbreak of 1992, 40 years later, created serious concerns about the survival of the cotton industry in the short-term without effective chemical controls. Local research discovered that putative pyrethroid resistances in the B-biotype of *B. tabaci* could be overcome with an appropriate synergist like acephate or endosulfan,^25^–^29^ much as in other parts of the world.^30^ During the early stages of this crisis, 1993–1995, growers depended on key pyrethroid mixtures such as fenpropathrin plus acephate and bifenthrin plus endosulfan.^29^ The consequences of the use of such broad-spectrum biocides and the dependence on their repeated use caused much concern within scientific, governmental and industry circles, which all collaborated to exchange information within an annual USDA program-planning process.^31^,^32^ These severe outbreaks were met with a similarly acute response by the research, extension and industry community, which ultimately resulted in the development and continual evolution of a highly effective and efficient pest management program reflective of the forward vision embodied in the ICC.

## THE ECOSYSTEM CONTEXT

**Figure 1:**
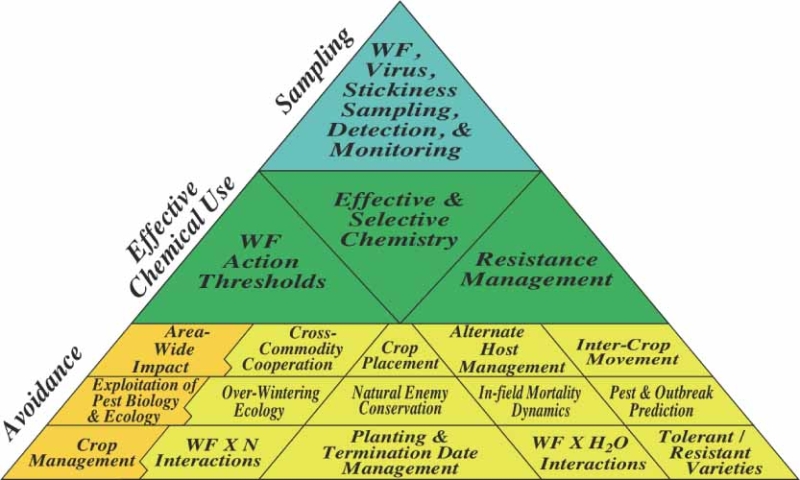
Conceptual diagram of IPM showing the main interacting management tactics arranged in an inherently stable pyramid where elements build upon one another resulting in a sustainable management strategy. Adapted with permission from Elsevier^68^.

A key underlying element of the ICC proposed by Stern *et al.*^1^ was ‘to recognize the “oneness” of any environment, natural or man-made’, and to understand that any control system imposed on a given pest in a given crop has consequences for the management of other pests and crops in the ecosystem. They also emphasized the multitactic nature of pest management, including consideration of factors such as plant nutrition, plant physiology and plant resistance and the economic considerations that bind them all. A proxy for this vision can be depicted in a pyramid representing the multiple tactics that can be brought to bear on a pest issue, as well as how these tactics interact, complement and build upon one another at different levels in an overall pest management strategy (Fig. 1). A large portion of the ecosystem as envisioned by Stern *et al.*^1^ is embodied in the foundational (avoidance) elements of the pyramid, such as area-wide impact, exploitation of pest biology and ecology and crop management. The pyramid also helps to demonstrate the simplicity, and at the same time the complexity, of the ICC, which calls primarily upon the interaction of four key elements: sampling, thresholds, chemical control and biological control, but as they are embedded in the overall ecosystem context.

### Pest population dynamics and demography

**Figure 2:**
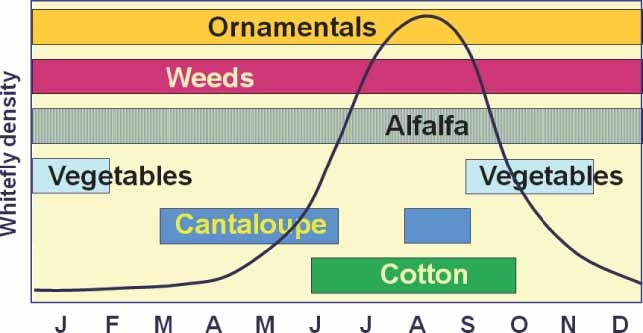
Depiction of a typical seasonal cycle for *B. tabaci* in an Arizona agroecosystem. The line shows the general pest population density over time.

**Figure 3:**
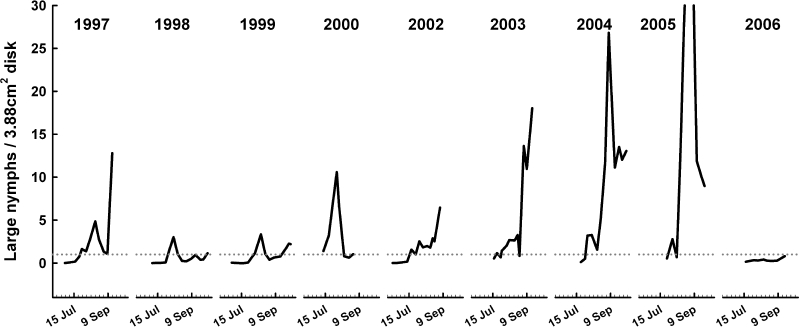
Population dynamics of *B. tabaci* over a ten year period in unsprayed fields at the University of Arizona, Maricopa Agricultural Center in south-central Arizona. The dotted horizontal line represents an action threshold of 1 large nymph (3^rd^ or 4^th^ instar) per quarter-sized leaf disk (3.88 cm^2^).

Many of the biological qualities of *B. tabaci* enable the insect to thrive in the low desert production areas of Arizona and southern California. The lack of any quiescence period allows the insect to develop and reproduce year round, limited only by temperature, the availability of suitable host plants and natural control elements in the environment. Watson *et al.*^33^ outlined the seasonal cycle of *B. tabaci* in an Arizona agricultural setting (e.g. Fig. 2). Populations persist at very low densities during winter months on winter vegetables, weeds and perennial hosts such as alfalfa and ornamentals within urban and rural landscapes, build on spring-planted crops such as cantaloupe (a favored host), reach peak densities during summer months on crops such as cotton and then decline during the fall. *B. tabaci* populations frequently exceed established threshold levels (see Section 4.1.1) in Arizona cotton (Fig. 3), although the magnitude and extent of these outbreaks is highly variable both over years and across the cotton production region of the state, much as depicted in the insightful schematic of Stern *et al.* (p. 90).^1^ Overall, the seasonal cycle is enabled by the insect's ready ability to disperse within the environment and continually exploit new hosts.^34^–^36^ These qualities make *B. tabaci* not just a single crop pest but an ecosystem pest, and this ultimately drives the formulation of pest management strategies on both local and regional scales.

**Figure 4:**
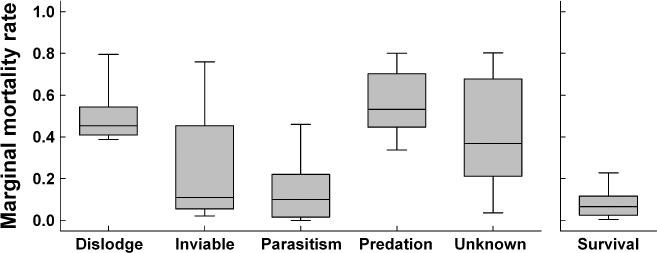
Box plots showing levels of marginal mortality of immature *B. tabaci* by various factors and overall generational survival in unsprayed cotton fields over 14 generations. The line within each box represents the median, the box bounds the 25th and 75th percentiles, and the whiskers denote the 10th and 90th percentiles. Adapted with permission from Wiley InterScience^37^.

**Figure 5:**
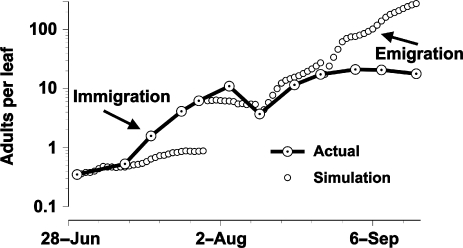
Comparison of simulated and actual population trajectories for adult *B. tabaci* in cotton. Simulation is based on a temperature-dependent, stage-structured model that was initiated with actual insect stage densities and observed rates of immature mortality from life tables. Adapted with permission from Wiley InterScience^37^.

Somewhat counterbalancing the huge biotic potential of *B. tabaci* has been the discovery that populations of this pest are subject to high levels of natural mortality (>93%), both in the cotton system^37^ (Fig. 4) and in a variety of crops and host plants that comprise the seasonal cycle of this insect.^38^ The sessile nature of the immature stages of this insect afforded the opportunity to directly and accurately measure rates of mortality by various factors via life table studies in the field. As detailed below, predation is the key factor determining intergenerational variation in *B. tabaci* mortality, and this mortality factor also contributes the highest level of irreplaceable mortality.^37^ Further life table studies on other crops and host plants of *B. tabaci* showed that predators also provide high levels of mortality of *B. tabaci* on ornamental lantana, alfalfa, spring and fall cantaloupe and various annual weeds.^38^ One key finding from this life table work in non-cotton host plants has been the discovery that total generational mortality in spring cantaloupes is relatively low (≈65%), which likely acts as a biological release valve during the late spring, precipitating large influxes of whiteflies into temporally and spatially adjacent crops such as cotton. The role of migration and dispersal in the population dynamics of *B. tabaci* has been acknowledged by researchers^39^–^43^ for many years, but there has yet to be any direct or quantitative measure of the phenomenon. However, comparison of simulated population dynamics, based on life table mortality rates to generate endogenous population growth of *B. tabaci*, with actual population densities in cotton hints at the magnitude of early-season immigration and late-season emigration^37^ (Fig. 5). This pattern of immigration into cotton plays a key role in the management strategy for this pest that is detailed later.

## THE MANAGEMENT SYSTEM

### Sampling and thresholds

**Figure 6:**
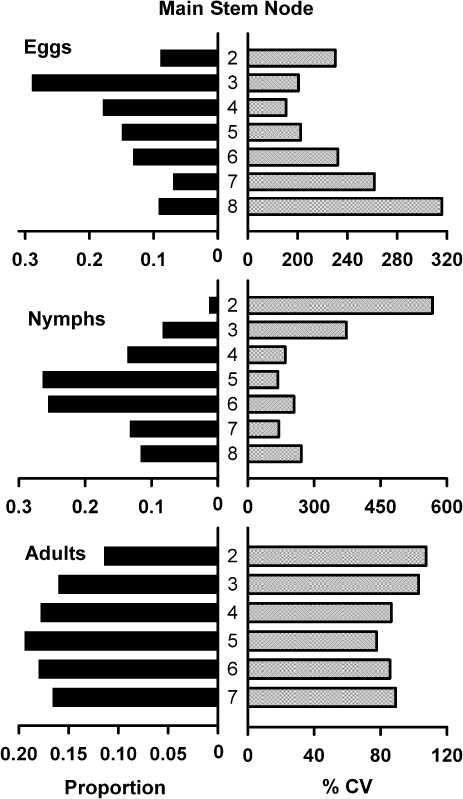
Distribution and variation of immature and adult stages of *B. tabaci* on mainstem node leaves of cotton. Based on these patterns and the accuracy of leaf-based counts to predict whole plant abundance, the 5^th^ node leaf was chosen as the optimal sample unit for estimating population density. Adapted from^47^,^49^.

Sampling is a fundamental tool for the study of population dynamics and the formulation of decision aids in a pest management program. This was one of the first tools developed upon the invasion of the new *B. tabaci* biotype into Arizona. Guided in part by prior work,^44^–^46^ basic information was developed on within- and between-plant distributions of immature stages, optimal sampling units were selected on the basis of variability and costs and fixed-precision sampling models and plans were developed and validated.^47^,^48^ Equivalent studies were conducted for the adult stage, with the added element of exploring different methods for estimating density of this mobile stage.^48^–^50^ The result of this work was the development of simple plans based on counting immature and adult stages on the underside of cotton main stem leaves located at the fifth node below the terminal (Fig. 6) that reflect densities on the entire plant. These sampling plans were instrumental in the study of basic population dynamics, in conducting experiments to test and evaluate various control tactics (e.g. references 51 to 54) and ultimately in the development of thresholds.^55^,^56^

#### Evolution and validation of decision aids

Perhaps the most critical and long-standing concept introduced by Stern and colleagues^1^ was that of the EIL, ‘the lowest population density that will cause economic damage’, and the associated ET, ‘the density at which control measures should be determined to prevent an increasing pest population from reaching the economic injury level’. Although these have since been defined in more specific mathematical terms amenable to experimentation and development,^57^,^58^ they remain the guiding principle of modern pest management based on prescriptive control. Stone and Pedigo^59^ were the first to develop an EIL, and since that time hundreds of EILs and ETs have been developed for a range of agricultural and horticultural commodities.^60^

The initial invasion of *B. tabaci* biotype B into Arizona happened suddenly, requiring a rapid response to provide producers with some basic tools for management. In addition to the development of sampling tools, several concurrent studies attempted to derive usable EILs and thresholds for decision-making.^51^,^55^ A multistate project was initiated in 1993 to develop action thresholds for *B. tabaci* in cotton for California, Arizona and Texas.^56^ The main chemical tools available were pyrethroids synergized primarily with organophosphates, and the target of decision-making was the adult stage. The EIL research pointed to levels of around 5–15 adults per leaf, based on cotton prices and control costs of the time.^55^ Field experiments based on the deployment of a series of candidate, predetermined threshold levels pointed to action threshold levels of around 5–10 adults per leaf.^51^,^56^ Further experience with typical population trajectories and concern about lint stickiness from honeydew contamination led to the selection of an economic threshold of five adults per leaf in Arizona and the desert valleys of California. This level preserved yield and lint quality while also permitting effective suppression with the chemical arsenal available at that time. In the San Joaquin Valley of California, where *B. tabaci* was less problematic, a threshold of ten adults per leaf was adopted.

Stern *et al.*^1^ further pointed to the critical need for sampling plans that were rapid and simple enough to use, so that consultants and growers would readily adopt them. Since the inception of ICC, sampling theory and practice have grown considerably,^61^–^63^ and hundreds of sampling plans have been developed for making treatment decisions relative to critical densities.

Based on the newly defined threshold, a simple binomial sampling plan for classifying the density of *B. tabaci* adults was developed,^64^ validated on over 3200 ha of commercial cotton,^48^ and delivered and taught to growers and pest control advisors through intensive and extensive workshops throughout the state.^65^–^67^ Although the original binomial model was developed as a sequential sampling plan, based on user input, it was delivered as a fixed-sample-size plan requiring that 30 leaves be examined per field (15 each at two sites in a field) before determining the need for control action. The whole decision process took about 5 min to execute and was very accurate, leading to the correct decision nearly 88% of the time.^48^

The decision protocol enabled a version of supervised control, was enthusiastically adopted by growers and helped them maintain profitability in the face of severe *B. tabaci* outbreaks in the early 1990s.^24^,^68^ However, this fell short of IC, because the broad-spectrum insecticides in use severely disrupted natural enemy populations and biological control played essentially no role.^69^ Not unexpectedly, overreliance on synergized pyrethroids led to rapid evolution in loss of susceptibility in *B. tabaci* populations by 1995,^29^,^70^ precipitating the need for a new strategy centered around buprofezin and pyriproxyfen, two insect growth regulators (IGRs) that had been successfully used in Israel for suppression of *B. tabaci* in cotton and greenhouse systems for many years^71^,^72^ (see Section 4.2.2). Both IGRs were shown to have low vertebrate toxicity,^73^,^74^ and numerous laboratory studies pointed to their putative selectivity in the field,^75^–^81^ which was later verified in the Arizona cotton-whitefly system^82^,^83^ (see Section 4.3).

Because these IGRs are mainly active on the immature stages of *B. tabaci*, the focus of the decision protocol was expanded to include both adult and immature stages. A provisional threshold of 0.5–1 large nymphs (third and fourth instars) per quarter-sized leaf disk was determined on the basis of experience with the IGRs in Israeli cotton (Horowitz AR, private communication, 1995) and patterns of seasonal population structure and growth in Arizona cotton. Additional experience in the field showed that densities of around 3–5 adults per leaf, coupled with evidence of in-field reproduction (via large nymphs), denoting the leading edge of an upward trajectory of population growth, represented the optimal time to deploy these IGRs. The objective was to initiate control with IGRs when nymphal populations were relatively low but at an inflection point leading to inevitable upward growth. A fixed-sample-size binomial sampling plan was developed for large nymphs using the leaf disk (3.88 cm^2^) as the sampling unit to facilitate efficiency.^84^ The dual-stage decision protocol was delivered with a matrix of decision outcomes depending on adult and nymphal densities.^66^ For example, if neither threshold was exceeded, resampling in 3–7 days was recommended, but, if nymphal densities exceeded the threshold and adults did not, then the decision was to resample in 3 days or apply buprofezin because of its effect only on nymphal stages. This matrix approach allowed some flexibility in decisions when growth of the two stages might be asynchronous owing to atypical immigration events (see above) or previous insecticide use. A commercial-scale study served to validate the decision protocol^85^ and showed that the nymphal sampling plan was accurate, leading to the correct decision with regards to insecticide applications nearly 89% of the time.^84^ The dual-stage sample plan and threshold takes about 7 min to execute in the field with a sample size of 30 leaves and remains the underlying strategy for decision-making while supporting additional selective and partially selective insecticide options that have since been identified and added^86^ (see Section 4.2.2).

### Chemical and biological control

Stern *et al.*^1^ articulated a clear vision for how IC could be accomplished: ‘Integrated control is most successful when sound economic thresholds have been established, rapid sampling methods have been devised, and selective insecticides are available’. At the time of the spotted alfalfa aphid outbreak in 1954, there was little understanding of, or value placed on, selective insecticides. Much like the invasion of *B. tabaci* in the early 1990s, the dimension of the crisis was such that immediate relief was needed in the form of effective chemical controls. Stern *et al.*^87^ stated: ‘There is little doubt that the insecticides parathion, malathion, and TEPP prevented a widespread devastation of California's alfalfa industry following the 1954 appearance of the spotted alfalfa aphid *Therioaphis maculata* (Buckton), but the materials also proved to be toxic to insect enemies of the aphid’.

Stern and his colleagues conducted a wide array of field tests in commercial alfalfa fields over a short period of time in the southern valleys of California.^87^–^89^ Their findings helped them identify demeton (Systox) as a compound with potential selectivity in their system. Even though parathion and other chemistries were at times nearly 100% effective, the occurrences of pest resurgence and potential for secondary pest outbreaks convinced them that the strategic use of a selective compound like demeton would better complement the existing biological controls present.^87^–^89^

These field studies provided the empirical basis for the ICC, and their views on the role of chemical control. They wrote: ‘with adequate understanding [chemical and biological control] could be made to augment one another’.^1^ More fundamentally, they defined a selective insecticide as one that, while killing the pest individuals, ‘spares much or most of the other fauna, including beneficial species, either through differential toxic action or through the manner in which the insecticide is utilized (formulation, dosage, timing, etc.)’.

#### Ecosystem services and conservation biological control

Sparing fauna of any kind in agricultural fields was not a common theme among pest management practitioners of the time. However, these themes pervade the IPM literature of today and are now guiding a new generation of ecological theory in the realm of ecosystem services. Daily^90^ defines ecosystem services as ‘the conditions and processes through which natural ecosystems, and the species that make them up, sustain and fulfill human life’. This concept has since been expanded to include managed systems and organized around four basic categories of ecosystem services: supporting, provisioning, regulating and cultural (e.g. reference 91). Stern *et al.*^1^ saw the importance of ‘regulating services’ in conservation biological control and stated that ‘… these regulating factors actually keep thousands of potentially harmful arthropod species permanently below economic thresholds’.

Conservation biological control is widely recognized as a key ecosystem service that provides target and secondary pest control while preventing pest outbreaks and resurgences. Many studies today are dedicated to manipulating the environment in strategic ways so as to maximize this regulating service to natural and managed ecosystems. Much effort has focused on conservation biological control enhancement via the use of alternative food sources including artificial sprays, provision of shelter, exploitation of semiochemicals and other aspects of habitat management (see reference 92 for a review). And, in spite of considerable research on defining toxicity of insecticides to natural enemies (e.g. reference 93), relatively little attention has been paid to manipulation of chemical controls towards compatibility with biological controls in the field, a foundational element of ICC. Arguably, chemical control is the principal tactic in use by most pest managers. The Millennium Ecosystem Assessment^91^ concluded that pesticide use around the world was diminishing these regulating services and in fact replacing pest control with natural enemies. It further concluded that pesticide use itself was degrading the ability of agroecosystems to provide for pest control.

#### Selectivity as the basis for integrated control

Like demeton 50 years ago in alfalfa, pyriproxyfen and buprofezin were the key chemical controls tested in the mid-1990s that provided an opportunity to more strategically manage *B. tabaci* in Arizona cotton (e.g. references 53 and 94 to 96). These IGRs had no previous registrations in the USA. Starting in 1996 under EPA Section 18 emergency exemption, growers had simultaneous access to these two compounds in response to the emergency need and outbreak of 1995. Large-scale testing, including aerial applications, was conducted,^68^,^85^,^97^ and new guidelines were developed and deployed^66^,^98^,^99^ (see Section 4.1).

Pyriproxyfen is a juvenoid with activity on developing eggs and metamorphosing nymphs. Its translaminar activity permits extended control even from aerial applications, in spite of very dense plant canopies and pest feeding behavior concentrated on the abaxial surface of leaves. Buprofezin is a chitin synthesis agonist with activity on *B. tabaci* nymphs. Its vapor activity is also important in the redistribution of residues within the plant canopy. Later, acetamiprid, a neonicotinoid with excellent acropetal movement in the plant, was registered in 2002 for Arizona cotton. Spiromesifen, a lipid biosynthesis inhibiting ketoenol, was registered in 2005. These four compounds have become the current-day chemical arsenal used to augment *B. tabaci* control in Arizona cotton.

The extension guidelines in use today^86^,^100^ are the product of progressive refinement of the original guidelines^66^ and a better understanding of the role of chemical and biological control in the Arizona agroecosystem. *Bemisia tabaci* chemical controls are divided into three stages defined not only by their efficacy but also by their selectivity attributes. Stage I contains all the fully selective compounds (pyriproxyfen, buprofezin and low rates of spiromesifen). Stage II contains partially selective compounds like acetamiprid and other neonicotinoids, or higher rates of spiromesifen. Stage III is reserved for the synergized pyrethroids that were once the mainstay of the chemical control program in the early 1990s. Today, these broad-spectrum options are recommended for use only in the late season, if needed, to assist in the control of *B. tabaci* along with other pest insects. These guidelines do not mandate a specific sequence or rotation among chemical use stages, but teach growers that more selective approaches will create more effective ecosystem services that will provide regulation of all pest species. The benefits of IC are regularly taught to growers and pest control advisors (PCAs), the licensed professionals who prescribe pesticide use in Arizona. Conditions are identified when greatest benefits may be gained by deploying stage-I chemistry first [e.g. typical build-up and balance (nymphs and adults) of populations (see Sections 3.1 and 4.1.1); use of transgenic Bt cotton for selective control of caterpillar pests and with no prior sprays for other insect pests; sufficient time to the end of the season to permit the functioning of the IGRs]. However, stage-I compounds in current use have no strong adulticidal action. Therefore, in scenarios of large immigrating populations, growers are encouraged to make use of the adult active neonicotinoids, especially acetamiprid, which has longer residual and better systemic characteristics. Landmark grower agreements and guidelines are also in place to manage resistance and to share chemistry among multiple crops, depending on cropping complexity.^29^,^100^,^101^

#### The importance of ecological context

The ecological context is critical to understanding the selective potential of any approach. While laboratory toxicological studies are valuable (e.g. references 102 and 103), they do not adequately address the milieu in which the candidate agent is to be used or the manner in which biological control agents interact with that environment. Initially alarming reports of centuries-old biological control being disrupted by new chemistry (e.g. cottony cushion scale controlled by Vedalia beetle in Californian citrus) have since been quelled by the more careful timing and application of pyriproxyfen in those systems.^104^,^105^ Nevertheless, the potential toxicological impact of these IGRs, particularly pyriproxyfen, on non-target coccinellids should be reconciled with the ecological context in which the materials are to be used.^106^–^108^ Thus, pyriproxyfen and buprofezin have proven quite selective in the Arizona cotton–*B. tabaci* system,^66^,^82^,^83^,^109^ but pyriproxyfen has shown potential conflict with biological control in citrus systems in California,^103^,^105^ Australia^108^ and South Africa.^107^ Even then, compounds with potential toxicity to natural enemies may fail to reach them in sufficient dosages to cause harm (e.g. only 15% of labeled pyriproxyfen reached *Podisus maculiventris* Say from its intoxicated prey).^102^,^108^

As an organophosphate, demeton was a broadly toxic compound with much potential for harm against certain natural enemies. However, Stern and colleagues' approach was to test as low a rate as possible to accomplish sufficient pest control with the greatest safety to beneficial organisms (for example, see Fig. 16). Even Smith *et al.*^108^ concluded that very low rates of pyriproxyfen in Australian citrus would control citrus red scale with some margin of safety for Vedalia and other coccinellid beetles. In another example, novaluron, a chitin-inhibiting IGR, has shown great potential for *B. tabaci* and other pest control in Israeli cotton.^110^ However, recent testing in the Arizona cotton system has shown it to be as destructive to natural enemies as the negative control, acephate, and more likely to contribute to pest resurgence of *B. tabaci* (Ellsworth PC and Naranjo SE, unpublished). As a consequence, this product is not recommended for use in cotton in Arizona, in spite of its availability since 2004.

In a remarkable statement for its time, Stern *et al.*^1^ warned that ‘… failure to recognize that control of arthropod populations is a complex ecological problem … leads to the error of imposing insecticides on the ecosystem, rather than fitting them into it’. For Arizona cotton, this suggests that novaluron is poorly suited for present needs because of its toxic effects on hemiptera and the reliance on generalist hemipteran predators for biological control (see Section 4.3). In comparison, pyriproxyfen used indiscriminately in a citrus system for scale control may disrupt biological control processes dependent on coccinellids there. However, lower dosages (<2 mg L^−1^)^108^ and more properly timed applications of pyriproxyfen in Californian citrus orchards have effected long-term control of citrus red scale and other scale insects without disruption of coccinellids and with better safety to hymenopteran parasitoids than the alternatives.^104^ Furthermore, the Arizona cotton system is not typically dependent on the ecosystem services provided by Coccinellidae, nor have negative effects been measured on this group of insects. On the contrary, Coccinellidae have generally been conserved in Arizona cotton compared with the conventional alternatives.^83^

#### Pesticides poison the concept?

Those familiar with the original ICC, or later its expansion to IPM, have often been frustrated by the course that these concepts have taken; so much so that alternative monikers have been developed over the last 20 years. There have been calls for biologically or ecologically intensive IPM, organic or sustainable pest management and intriguing spin-offs such as integrated biodiversity management (IBM).^111^ There are also indictments that IPM has become nothing more than integrated pesticide management,^112^ leading to modifiers like ‘real’ or ‘true’ being added to IPM. At the core of many of these critical shifts is a genuine frustration with the use of pesticides in IPM. However, the ICC was genius in this regard by acknowledging the role that chemical control plays in systems and noting that agricultural production of any scale is a distortion of natural processes and therefore unlikely to be sustained by regulating ecosystem services alone. Furthermore, ‘provisioning’, or the production of food and fiber for society, is a key ecosystem service that needs to be put in balance and perspective with other ecosystem services. Denial of this fundamental ecosystem service (i.e. provisioning) will only serve to alienate and confuse the practitioner and producer who simply wish to control pest populations in the least disruptive manner possible while producing a safe and abundant food supply.

The role that chemical control plays in modern-day IPM is subject to both scientific skepticism and overzealous marketing. Suppliers of pesticides are very savvy and have capitalized on the conceptual discord present by selling nearly everything as reduced-risk, biorational (see reference 113) or ‘soft’ on beneficials. Cleverly placed lacewings or other beneficial archetypes adjacent to a chemical jug or plant have now become the norm in the marketing of any new insecticide. At some level, this supports the idea that the ICC has penetrated the agricultural industry psyche; however, the claims made for these chemicals are rarely, if ever, subject to the important research that is needed to validate their selectivity within the relevant agroecosystem context.

### Bioresidual, biorational and validated integrated control

**Figure 7:**
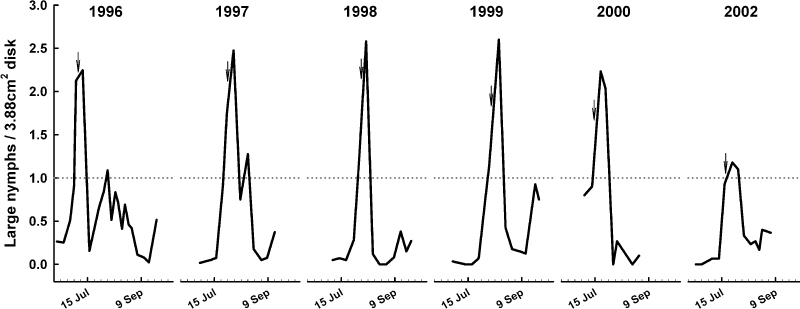
Population dynamics of *B. tabaci* over a six year period in IGR-treated fields at the University of Arizona, Maricopa Agricultural Center in south-central Arizona. The dotted horizontal line represents an action threshold of 1 large nymph (3^rd^ or 4^th^ instar) per quarter-sized leaf disk (3.88 cm^2^). Populations increased past threshold, were treated once with an IGR, and after a short delay, collapsed and remained sub-economic in most years (also see Fig. 3). Arrows denote the timing of insecticide application.

**Figure 8:**
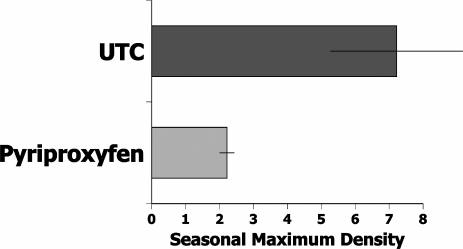
Seasonal average maximal density ( ± SEM) for large nymphs (3^rd^ or 4^th^ instar) per quarter-sized leaf disk (3.88 cm^2^) over five years of replicated, large-scale assessments of IGRs in comparison to the UTC in cotton, Maricopa, Arizona, USA (*P* = 0.02). The properly timed usage of IGR sprays significantly lowered the maximal seasonal density of whiteflies compared to the UTC. Severe economic loss (reductions in yield and quality) is common at levels in excess of 4 large nymphs per disk^86^.

Experiences with the IGRs in large-scale replicated and unreplicated experiments since 1996 have shown a very consistent pattern: pest population increase to and through the economic threshold (see Fig. 3), an IGR spray is applied, continued short-term growth in the population ensues, drastic population collapse occurs and eventual long-term suppression and subeconomic levels of *B. tabaci* are achieved (Figs. 7 and 8). This extended suppressive interval made possible by the use of a selective insecticide was coined ‘bioresidual’.^68^,^114^ It is defined as the ‘combined contribution of all natural mortality factors … that allow for lowering of the general equilibrium position of the target pest and long-term pest control following the use of selective insecticides that is otherwise absent with most broad-spectrum insecticide use’.^109^ The goal in developing this term was better to communicate to growers the potential selectivity benefits of their control chemistry and to accommodate all the mortality processes present in a selective system, especially those related to conservation biological control. Stern *et al.*^1^ defined natural control as the combined actions of abiotic and biotic elements of the environment. Bioresidual is simply that natural control possible when a selective insecticide is used in a well-timed and effective manner, providing extended suppression that has been measured up to 12 weeks in the cotton/whitefly ecosystem. This regulating ecosystem service helps form the foundation our IPM system (Fig. 1).

Designing and labeling a product as selective or ‘biorational’ is not sufficient, however, to establish its selectivity.^113^ Some effort must be made to validate the candidate approach or product in the system of interest, because, as noted, the control dynamic is embedded in an ecological context. A product may be fully selective in one environment and catastrophically disruptive in another. Laboratory and other bioassays without an ecological context are inadequate for establishing insecticide selectivity in a system.

There are several ways to verify and validate an approach as being selective. General observation can establish the presence and function of natural enemies. Natural enemy densities can be examined in comparative systems using innovative community ordination methods (e.g. principal response curves).^115^,^116^ The functional role of natural enemies and other mortality factors can further be inferred from predator–prey dynamics and demography. The Arizona system is based on this kind of careful research and validation, which are key to the development and implementation of IC, the unique interplay between chemical and biological control that is fully maximized by a validated decision support system (Fig. 1).

**Figure 9:**
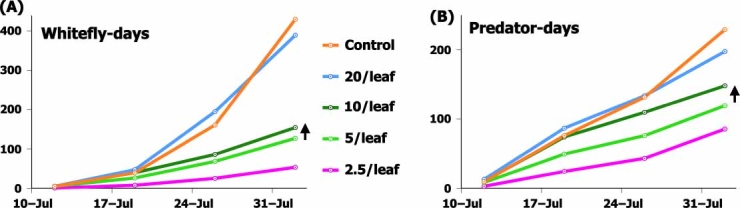
**(A)** *B. tabaci* population dynamics in cumulative whitefly-days (adults per leaf) and **(B)** predator population dynamics in cumulative predator-days (total counts of 12 species of predators per 25 sweeps), for five nominal action thresholds (adult whiteflies per 5^th^ mainstem leaf) for conventional chemistry and an UTC in cotton, 1994, Maricopa, Arizona, USA. There were significant treatment effects on every date (*P* < 0.05). Arrows show small and somewhat larger increases in whitefly and predator densities, respectively, for two candidate thresholds, suggesting benefits in conservation biological control by deferring treatments until 10 adults per leaf. Re-drawn from^69^.

Early work carried out in the 1950s, even before the availability of putative selective insecticides, showed that selectivity could be accomplished in part through effective timing of insecticide applications. Naranjo *et al.*^69^ showed that some changes in adult thresholds for conventional materials could have relatively minor effects on *B. tabaci* control dynamics, yet relatively large impacts on the associated predator dynamics (Fig. 9). They concluded that more potential for biological control was possible if producers deferred chemical controls until ten adults per leaf instead of five adults per leaf. Lower thresholds were too expensive to maintain, and higher ones were insufficient to prevent economic loss (see Section 4.1). They further concluded that the date of first spray was a key factor in predator dynamics in a system dependent on broad-spectrum chemistry.

#### Natural enemy community responses

**Figure 10:**
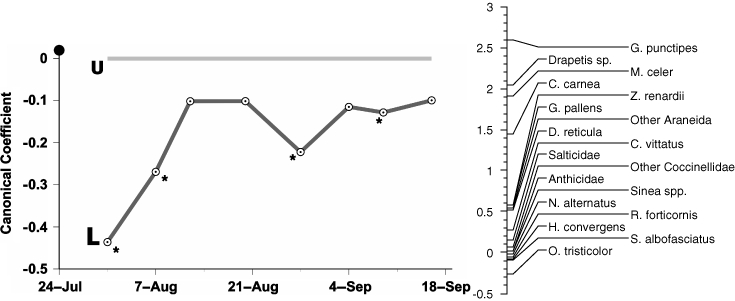
Principal response curves (PRC) showing the long-term negative effects possible from a single broad spectrum Lygus insecticide [(L) = acephate spray] on the predatory arthropod community comprising of ca. 20 taxa in cotton, 1997, Maricopa, Arizona, USA. Species weights indicate strength of the trend for each species or species group, with values > 0.50 generally reflecting the trends shown. Weights < −0.50 indicate a negative association or inverse of the trends depicted. The product of species weight and the canonical coefficient (y-axis) for a given treatment and time equals the natural log change in density of that species relative to the control. Dot at top of y-axis denotes timing of an acephate spray; Stars, denote significant differences between the acephate treatment and the UTC (y = 0) by date (*P* < 0.05). The PRC analysis over all dates was significant (*P* < 0.01) based on an F-type permutation test. Adapted with permission from Elsevier^83^.

Principal response curves (PRCs), a multivariate, time-dependent, analytic approach,^115^,^116^ present a powerful way to visualize and understand whole-community responses to external treatments via canonical coefficients and species weights. Canonical coefficients are a scaled measure of species densities relative to some standard (usually an untreated check), while species weights denote the correspondence of each species to the overall community pattern. Species with weights greater than 0.5 are generally considered most influential and most reflective of the estimated PRC pattern. Species with weights lower than −0.5 are also considered to influence the trends shown, but in the opposite direction. When a broad-spectrum insecticide is used, for example, dramatic and immediate reductions in natural enemy densities occur compared with an untreated check (UTC)^83^ (Fig. 10). Likewise, effects from a single broad-spectrum spray in cotton to control *L. hesperus* had significant negative consequences for the beneficial arthropod community for up to 7 weeks.

**Figure 11:**
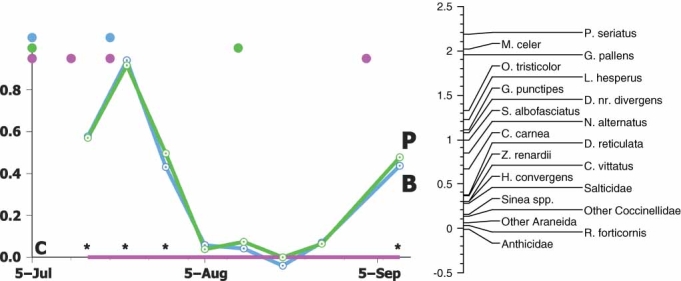
Principal response curves (PRC) showing the effects of different whitefly control strategies on the predatory arthropod community comprising of 20 taxa in cotton, 1996, Maricopa, Arizona, USA. Species weights indicate strength of the trend for each species or species group, with values > 0.50 generally reflecting the trends shown. Weights < −0.50 indicate a negative association or inverse of the trends depicted. The product of species weight and the canonical coefficient (y-axis) for a given treatment and time equals the natural log change in density of that species relative to the control. C = a rotation of conventional chemistry (control); P = pyriproxyfen-initiated rotation of IGRs; B = buprofezin-initiated rotation of IGRs; Dots denote timing of sprays of chemistry with corresponding color; Stars, denote significant differences between either IGR treatment and the rotation of conventional insecticides (y = 0) by date (*P* < 0.05). The PRC analysis over all dates was significant (*P* < 0.01) based on an F-type permutation test. Adapted with permission from Taylor & Francis^82^.

As part of a very large, commercial-scale evaluation, Naranjo *et al.*^82^ compared IGRs with conventional rotations of broad-spectrum chemistry commonly used at the time. Regimes initiated with either IGR generally supported more natural enemies than the conventional control, with a crab spider, *Misumenops celer* Hertz, among the top influencers of that PRC (Fig. 11). The only departure from the overall trend was when a commercially required spray for *L. hesperus* was needed over the entire experiment and a monsoon-associated storm impacted on the study area. Fewer sprays were needed in the IGR regimes for comparable control as a result of what was later learned to be the bioresidual present.

**Figure 12:**
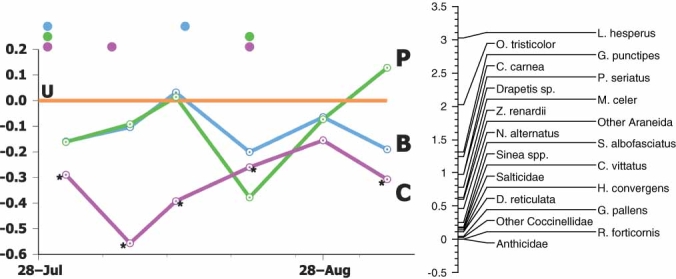
Principal response curves (PRC) showing the effects of different whitefly control strategies on the predatory arthropod community comprising of ca. 20 taxa in cotton, 1997, Maricopa, Arizona, USA. Species weights indicate strength of the trend for each species or species group, with values > 0.50 generally reflecting the trends shown. Weights < −0.50 indicate a negative association or inverse of the trends depicted. The product of species weight and the canonical coefficient (y-axis) for a given treatment and time equals the natural log change in density of that species relative to the control. U = UTC (control); C = a rotation of conventional chemistry; P = pyriproxyfen-initiated rotation of IGRs; B = buprofezin-initiated rotation of IGRs; Dots denote timing of sprays of chemistry with corresponding color; Stars, denote significant differences between adjacent treatment and the UTC (y = 0) by date (*P* < 0.05). The PRC analysis over all dates was significant (*P* < 0.01) based on an F-type permutation test. Adapted with permission from Elsevier^83^.

**Figure 13:**
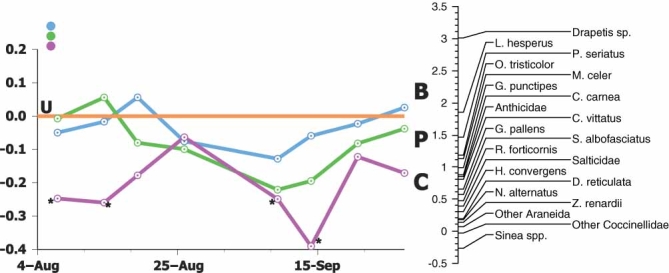
Principal response curves (PRC) showing the effects of different whitefly control strategies on the predatory arthropod community comprising of ca. 20 taxa in cotton, 1998, Maricopa, Arizona, USA. Species weights indicate strength of the trend for each species or species group, with values > 0.50 generally reflecting the trends shown. Weights < −0.50 indicate a negative association or inverse of the trends depicted. The product of species weight and the canonical coefficient (y-axis) for a given treatment and time equals the natural log change in density of that species relative to the control. U = UTC (control); C = a rotation of conventional chemistry; P = pyriproxyfen-initiated rotation of IGRs; B = buprofezin-initiated rotation of IGRs; Dots denote timing of sprays of chemistry with corresponding color; Stars, denote significant differences between adjacent treatment and the UTC (y = 0) by date (*P* < 0.05). The PRC analysis over all dates was significant (*P* < 0.01) based on an F-type permutation test. Adapted with permission from Elsevier^83^.

**Figure 14:**
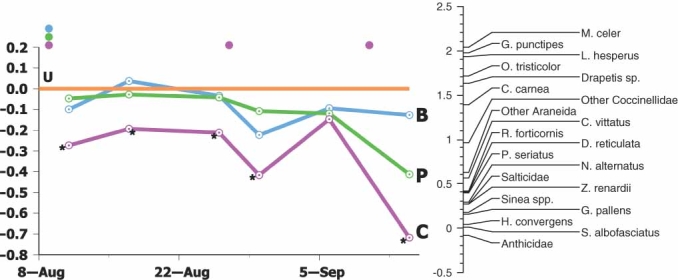
Principal response curves (PRC) showing the effects of different whitefly control strategies on the predatory arthropod community comprising of ca. 20 taxa in cotton, 1999, Maricopa, Arizona, USA. Species weights indicate strength of the trend for each species or species group, with values > 0.50 generally reflecting the trends shown. Weights < −0.50 indicate a negative association or inverse of the trends depicted. The product of species weight and the canonical coefficient (y-axis) for a given treatment and time equals the natural log change in density of that species relative to the control. U = UTC (control); C = a rotation of conventional chemistry; P = pyriproxyfen-initiated rotation of IGRs; B = buprofezin-initiated rotation of IGRs; Dots denote timing of sprays of chemistry with corresponding color; Stars, denote significant differences between adjacent treatment and the UTC (y = 0) by date (*P* < 0.05). The PRC analysis over all dates was significant (*P* < 0.01) based on an F-type permutation test. Adapted with permission from Elsevier^83^.

Subsequent large-plot replicated assessments, including UTCs, showed similar trends;^83^ only the major drivers of the relationships changed. In 1997, the sucking predators *Orius tristicolor* White and *Geocoris punctipes* Say were the predominant species involved (Fig. 12). In 1998, an empidid fly, *Drapetis* nr. *divergens* Loew, had the largest species weight (Fig. 13). This small fly preys on adult *B. tabaci* as an adult.^117^ While not a specialist, strictly speaking, it is often associated with the presence of adult *B. tabaci* in cotton. A comparable species is present in Israeli cotton and may play a similar role there, where *B. tabaci* became a key pest in the mid-1980s (*D. subaenescens* Collin).^118^ The 1999 study was driven by a combination of the species already mentioned (Fig. 14). Some other predators also play significant roles in these analyses. Chewing predators like *Collops vittatus* Say were important in two of the four years examined (1996–1999). Coccinellids are not typically present in mid-summer cotton in large numbers in Arizona, but a complex of species was important in one of the four years (Fig. 14). *Chrysoperla carnea* s.l. Stephens also had species weights greater than 0.5 in each year studied, but generally lower than those of the other sucking predators in the system. The larger predators present (e.g. *Zelus renardii* Kolenarti in 1997, *Nabis alternatus* Parshley in 1996, and spiders in 1997 and 1999) (Figs 11 to 14) may function more as intraguild predators on the primary predators of *B. tabaci* in the system.

The idea that different species dominate the community analyses in different years or locations in Arizona cotton is a remarkable testament to the complexity and plasticity of the cotton arthropod food web. Certain conditions may favor certain pathways in certain years and other pathways in other years. Using a species weight > 1 and omitting the primarily plant-feeding mirids (*Lygus hesperus, Pseudatomoscelis seriatus* Reuter), four, three, three and five species dominated the food webs in 1996–1999 respectively. These were the sucking predators *Geocoris* spp. (usually *punctipes*, but also *pallens*), *O. tristicolor* and *C. carnea*, the empidid *D*. nr. *divergens*, and the crab spider *M. celer*.

#### Biological control function

**Figure 15:**
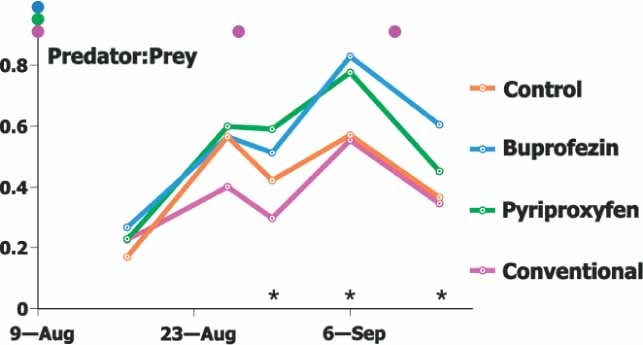
Predator–prey dynamics (as ratio of total counts of ca. 20 species of predators per 50 sweeps to total whiteflies per leaf) for three different whitefly control strategies and an UTC (Control) in cotton, 1999, Maricopa, Arizona, USA. Dots denote timing of sprays of chemistry with corresponding color; Stars, denote significant treatment effects by date (*P* < 0.05). IGRs increase the ratios favoring more efficient pest control. Adapted with permission from Elsevier^83^.

The function of this complex of predators can also be inferred by their relative abundance in these otherwise well-controlled field experiments. For example, the marginal decline in overall predator abundance, as depicted in PRCs, in the IGR-initiated regimes relative to UTCs is suggestive of some weak density dependence in relation to the most abundant prey^83^ (Figs. 11 to 14). Predator to prey ratios are another comparative method for inferring predator function. Naranjo *et al.*^83^ constructed ratios based on all predators captured in 50 sweeps and all *B. tabaci* per leaf found in cotton. The ratio increased initially in the UTC cotton, but then plateaued in what was an outbreak population of *B. tabaci* (Fig. 15). When conventional chemistry was used repeatedly to maintain control of *B. tabaci* populations, the predator:prey ratios were similar to, but lower than, the UTC, suggesting that both predator and prey were reduced equally. However, after initially similar ratios, the IGR-initiated regimes eventually resulted in higher predator:prey ratios 3–5 weeks after application. This suggests that the chemical residual of the selective IGRs was providing suppressive control initially, but that natural enemies were playing a large role in the bioresidual, thereby providing effective pest control extending to the end of the season.

**Figure 16:**
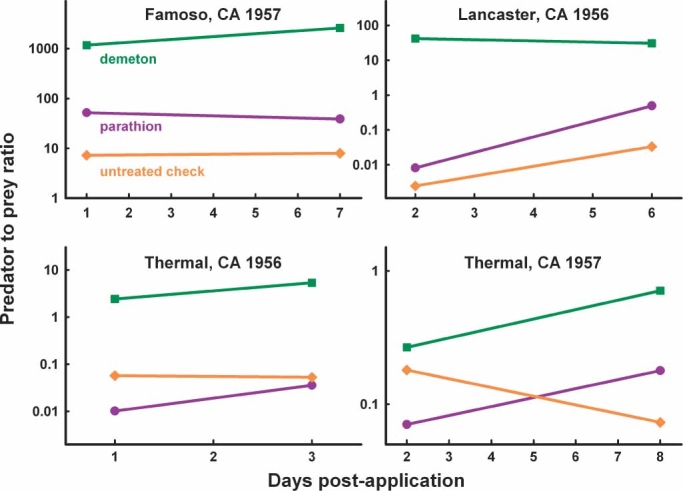
Predator–prey dynamics (as ratio of total counts of predators using various methods to spotted alfalfa aphids per stem) for two different aphid control strategies and an untreated check in alfalfa, 1956–1957, California, USA. Demeton use was associated with dramatic increases in the ratios, up to 3 orders of magnitude larger than the conventional standard, parathion. Data derived from published reports^87^,^89^.

Predator:prey ratios that favor pest control are very much in keeping with the vision of Stern *et al.*;^1^ ‘The ideal material is not one that eliminates all individuals of the pest species … [It] is the one that shifts the balance back in favor of natural enemies’. Interestingly, in spite of all their work with spotted alfalfa aphid and their many commercial evaluations of predator and prey abundances, Stern and coworkers never analyzed the basic relationship of predator to prey ratios. There was a huge advantage to the use of demeton compared with either parathion or the UTC (Fig. 16). The UTC supported outbreak levels of aphids. Both parathion and demeton effectively controlled the pest, but the latter was less harmful to the natural enemies, sometimes leading to a situation where abundant coccinellids had insufficient prey and even resorted to cannibalism.^89^ Nonetheless, demeton-treated areas consistently supported much higher predator:prey ratios and achieved better compatibility between chemical and biological controls.

Like parathion in the alfalfa system of 50 years ago, conventional sprays lowered prey densities as well as predator densities in the Arizona system. Furthermore, like Stern's demeton, the IGRs not only reduced prey numbers effectively, they also conserved existing predator numbers and created a more favorable balance of predators to prey. This more efficient control system should create collateral benefits in regulation of other pests in the system.

#### Validating biological control function through demography

**Figure 17:**
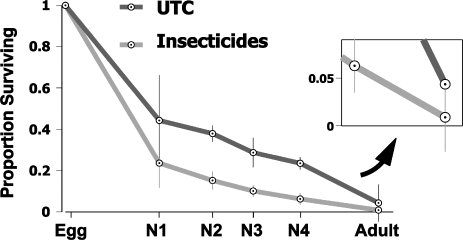
Average survivorship ( ± SEM) for 14 summer field generations of *B. tabaci* for IGR-initiated regimes (Insecticides) and an UTC (Untreated) in cotton, 1997–1999, Maricopa, Arizona, USA. Inset, blown-up view of survivorship to the adult stage. Only ca. 4% absolute difference in survivorship to the adult stage separates outbreak levels of whiteflies from well-controlled populations^37^,^109^.

To gain a better understanding of how survivorship of *B. tabaci* was changing in IGR-treated systems, it was necessary to use techniques of demography, specifically life tables. Naranjo and Ellsworth^37^,^109^ tracked 14 summer generations of treated and untreated *B. tabaci* over multiple years. The resulting survivorship curves for the two systems are striking in how similar the endpoints appear (Fig. 17). Few *B. tabaci* survive in cotton, even when not treated with insecticides, on average just ca 4.4%. However, when IGRs are used, not only is the shape of the curve changed, i.e. more *B. tabaci* die sooner in their life cycle, but fewer than 1% on average survive. Thus, pest managers are trying to leverage, on average, only about 4% absolute change in survivorship by using insecticides.^109^

Life table data can be used to calculate marginal mortality rates and, from this, irreplaceable or indispensable mortality, which is that portion of the total generational mortality that would not occur if a given mortality factor were eliminated.^119^ What happens when a mortality source fails to function? Is it replaced with contemporaneous factors? The Millennium Ecosystem Assessment^91^ concluded: ‘In many agricultural areas, pest control provided by natural enemies has been replaced by pesticides’. This is the antithesis of IC, where the two tactics fail to augment each other.

**Figure 18:**
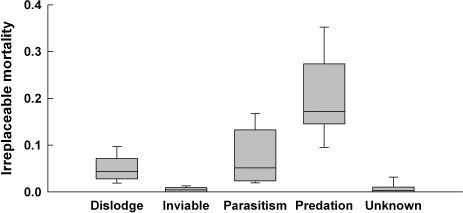
Box plots showing levels of irreplaceable mortality of immature *B. tabaci* by various factors in unsprayed cotton fields over 20 generations, 2000–2003, Maricopa, Arizona, USA (Naranjo SE, Ellsworth PC and Cañas L, unpublished). The line within each box represents the median, the box bounds the 25th and 75th percentiles, and the whiskers denote the 10th and 90th percentiles. Predation is the major mortality factor operating in untreated cotton.

In untreated cotton, Naranjo and Ellsworth^37^ calculated the largest net reproduction in *B. tabaci* when predation or dislodgement (a factor related in part to predation and in part to weather) was excluded from their analyses of irreplaceable mortality (see Fig. 4). They concluded that conserving predators and managing immigration of adult *B. tabaci* best achieved efficient pest management (see Fig. 5). Similar life table studies over 3 years in untreated cotton embedded in a multihost design showed very similar trends (Fig. 18) (Naranjo SE, Ellsworth PC and Cañas L, unpublished), with predation providing the largest proportion of irreplaceable mortality in *B. tabaci* populations. In all cases (7 years in total), parasitism has not been influential in *B. tabaci* control in cotton, in spite of rather significant shifts in the parasitoid fauna in Arizona.^120^

**Figure 19:**
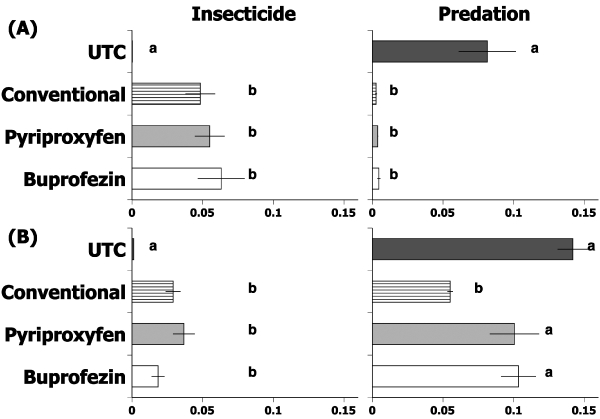
Irreplaceable mortality ( ± SEM; N = 12) of immature *B. tabaci* by insecticides or predation for three different whitefly control strategies and an UTC during **(A)** the first generation post-spray and **(B)** the second generation post-spray, 1997–1999, Maricopa, Arizona, USA. IGRs enable conservation biological control in the generation following application comparable to what is present in the UTC and contribute to their bioresidual. Adapted with permission from Elsevier^109^.

When insecticides are used in cotton, the relative importance of mortality factors is similar, but the relationship between insecticide-induced mortality and predation changes over time.^109^ Life tables constructed from the generation of *B. tabaci* present during the initiation of chemical controls were contrasted to life tables constructed from a subsequent generation, 3–6 weeks after the initial use of either IGR or conventional insecticides (Fig. 19). During the first generation of exposure, irreplaceable mortality supplied by insecticides or predation is similar no matter what compound is used. Either regime kills *B. tabaci* and is significantly different from the UTC. This shows how an effective insecticide supplants the irreplaceable mortality contribution of predation (Fig. 19A). Looking at the next time course (Fig. 19B), there is still significant, but much lower, insecticide mortality. Irreplaceable mortality due to predation, however, increased significantly where IGRs were used, now similar to the UTC. This shows mechanistically how one component of bioresidual, predation, is fostered by the use of selective insecticides.

#### Validation by disabling biological control

The final step in verifying that IC is functioning is to attempt to disable it. One obvious way to test this is to remove the chemical control agent and measure what happens. In years of testing, the UTC typically suffers serious deposits of whitefly-excreted sugars that make the marketing of the cotton impossible, and sometimes cause yield losses. However, this fails to demonstrate the compatibility of chemical and biological controls; only that chemical control is necessary to accomplish commercial production. Thus, the approach must involve disabling the biological control agents present in the system, usually through chemical exclusion.

**Figure 20:**
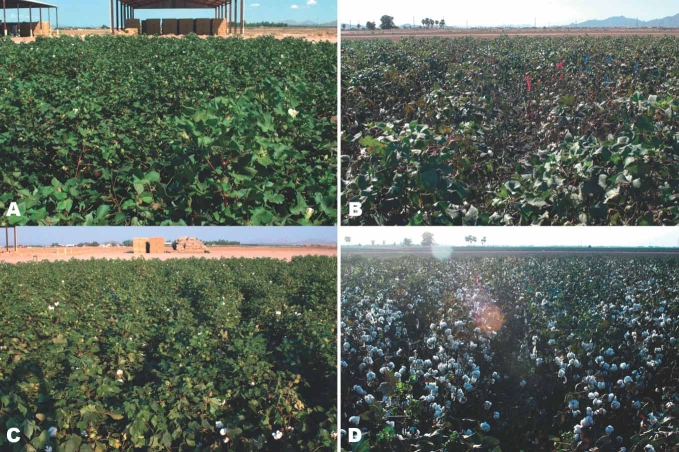
Photos of representative plots on two dates, 16-Sep (A, B) and 30-Sep (C, D) showing progressive damage resulting in premature defoliation and plant death in cotton by whiteflies released from control by four bi-weekly sprays of a broad spectrum insecticide (acephate; B, D) commonly used for Lygus control in comparison to UTCs (A, C), 2008, Maricopa, Arizona, USA.

In a recent study of irrigation and natural enemy effects, our graduate student excluded natural enemies in replicated plots using acephate on a biweekly basis for a total of four sprays (Asiimwe P, Ellsworth PC and Naranjo SE, unpublished). Acephate has poor efficacy against *B. tabaci* in Arizona cotton (Ellsworth, unpublished data), but is broadly toxic to a wide range of natural enemies and routinely used for *L. hesperus* control in Arizona.^121^ No other sprays were made. In this field experiment, regardless of irrigation regime, the acephate-treated plots sustained heavy damage from *B. tabaci*, including excess sugars, extensive sooty mold development and premature defoliation and plant death (Fig. 20). The UTC sustained much lower *B. tabaci* populations and the plants continued to grow normally.

In another series of studies to investigate candidate chemistry for *L. hesperus* control, biweekly sprays of acephate, in contrast to metaflumizone and flonicamid, were implemented for a total of three sprays in cotton. Community analyses showed severe and season-long depression of ca 20 non-target species where acephate was used. Furthermore, during years (2006–2007) when populations were at historically low levels otherwise, *B. tabaci* outbreaks were evident in the acephate-treated plots after the second application. No *B. tabaci* chemical controls were necessary in the other plots, and flonicamid and metaflumizone appear to be excellent selective controls for *L. hesperus* (Ellsworth PC and Naranjo SE, unpublished). This discovery fueled the widespread adoption of flonicamid, once it became commercialized and widely available, starting in 2007. More than half of all *L. hesperus* sprays in 2007 and more than 91% of all fields treated for *L. hesperus* in 2008 were sprayed at least once with flonicamid in central Arizona cotton (Ellsworth PC, unpublished data).

The relationship between control of *B. tabaci* and control of *L. hesperus* is a not a new one, as *L. hesperus* has been the number-one, yield-limiting pest of cotton since 1997 in Arizona.^121^,^122^ Ironically, it is in part because of the success of other selective controls for *B. tabaci* control (IGRs) and for lepidopteran control (Bt cotton) that *L. hesperus* has gained in prominence. Furthermore, the idea that chemical controls for one pest could interfere with the biological control of another pest is well known by practitioners. Stern and colleagues^1^ wrote: ‘Where prophylactic treatments are proved to be necessary for a perennial pest, selective materials must be developed and utilized to foster biological control both of other pests and of the pest of direct concern at other times’.

### Impact and outlook for IC and IPM

Some might say that IPM as a concept has left IC behind, and introduced new depths of complexity that the ICC did not readily accommodate (e.g. reference 123). However, given that arguably most IPM programs depend on some remedial chemical controls, IC could and should still exist at the center of the IPM concept (e.g. Fig. 1). Recalling the ecosystem nature of the Arizona system and its pests, the multidimensional elements of the Arizona IPM system owes its success to pest-source reduction and cross-commodity agreements in cotton, vegetables and melons,^29^,^100^ an unprecedented scale of host plant resistance in widespread transgenic Bt cotton deployment for pink bollworm control, IC of *B. tabaci* using fully or partially selective insecticides and most recently the availability and widespread usage of selective insecticides for *L. hesperus* control.

The results have been impressive and include an areawide change in the agroecosystem. As noted, Arizona's *B. tabaci* are shared among cotton, melon and vegetable crops that serve as year-round crop islands (Fig. 2). The cotton situation here has been discussed, but the outbreak of 1991/1992 nearly wiped out the fall melon and vegetable industry in Arizona and southern California. Emergency exemptions were sought for the soil use of imidacloprid. Palumbo (unpublished data) set up a series of replicated studies of soil use in commercial lettuce fields and compared *B. tabaci* dynamics in treated and untreated plots. The control was impressive, but the immigrating pressure was nearly overwhelming in 1993, and the UTC contained > 8 large nymphs cm^−2^. The industry adopted this soil usage more widely in 1994–1995, and areawide pressure in the UTC was lowered by an order of magnitude (ca 1–1.6 large nymphs cm^−2^), implicating a broad-area regional effect on *B. tabaci* population dynamics. Once the IGRs were deployed in 1996 in the cotton system, another order of magnitude lowering of *B. tabaci* densities was observed in the UTC over nearly a decade (ca 0.1 large nymphs cm^−2^). The combination of source reduction in fall melons and vegetables and IC of *B. tabaci* in cotton helped produce a decade-long period of areawide suppression of *B. tabaci* densities in the agroecosystem. Notable are new concerns (2006–2008) about lost performance in the imidacloprid–*B. tabaci* system where fall vegetables are grown (ca 50% reduction) (Palumbo JC, unpublished data).

**Figure 21:**
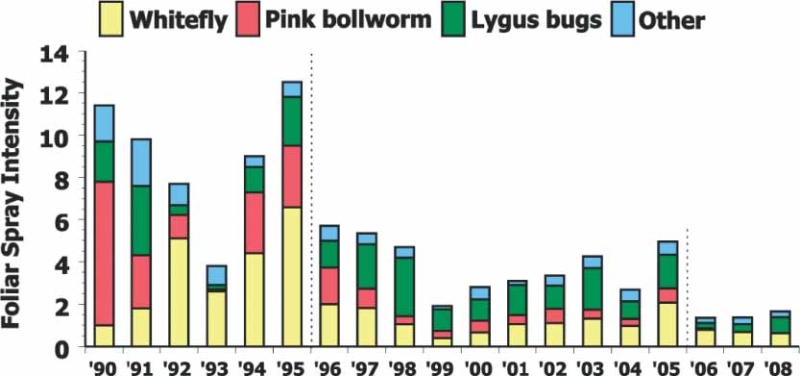
Statewide average foliar insecticide intensity (∼ sprays) made in cotton for key pests over time, 1990–2008, Arizona, USA. The IGRs, pyriproxyfen and buprofezin, and transgenic Bt cotton were introduced in 1996; pink bollworm eradication program efforts began in 2006 and flonicamid for Lygus control became available in 2006–2007 (derived from^124^).

The impact on grower practices, economics, health and environmental risks has also been historic. As part of an ongoing cotton survey process, the number and costs of cotton insect control in Arizona are determined annually.^124^ Cotton growers in Arizona were accustomed to spraying 5–10 times per season, starting in the 1990s (Fig. 21). *Bemisia tabaci* broke out statewide in 1992, and the majority of the control was focused on this pest using very broad-spectrum pyrethroids, organophosphates and endosulfan. The following year (1993) was filled with uncertainty, and growers curtailed their season by more than 30 days in order to minimize the damage caused by *B. tabaci* and the costs for control. The synergized pyrethroids became the control standards of the time (1993–1995), but, as noted, resistance threatened the continued use of these broad-spectrum mixtures and helped create outbreak conditions in 1995. In 1996, the IGRs were introduced, along with an extensive educational program to support the usage of these novel chemistries with decision support tools like sampling and thresholds.^68^ Transgenic Bt cotton for pink bollworm control was also introduced that year, but adopted on a minority of acreage. Adoption of Bt cotton increased to 60–80% over the next decade, until a pink bollworm eradication program was implemented and stimulated near-complete adoption starting in 2006 (95–98.3%). In 2008 there were no grower-initiated foliar insecticide sprays to control pink bollworm for the first time since 1965. Eradication program sprays against pink bollworm were made on fewer than 200 ha statewide (Antilla L, private communication, 2008). Selective chemistry for *L. hesperus* (i.e. flonicamid) became widely available in 2007 and led to acephate falling from its decade-long position as one of the top two most frequently used insecticides in Arizona cotton (Ellsworth PC, unpublished).

The number of sprays for *B. tabaci* declined to ca one spray per season shortly after the introduction of the IGRs. Environmental conditions and other factors in 2005 led to outbreak conditions for *B. tabaci* once again, but sprays and control costs were still relatively restrained. Growers sprayed, on average, 4.1 times to control *B. tabaci* prior to the introduction of the IGRs and after the invasion of the B-biotype of *B. tabaci* (5 years, 1991–1995). In contrast, growers sprayed 1.18 and 1.25 times in the 5 and 10 year post-IGR introduction intervals respectively. Even wider deployment of Bt cotton, starting in 2006 with pink bollworm eradication efforts and introduction and adoption of flonicamid as the principal *L. hesperus* control agent, has driven foliar insecticide usage to 30 year lows, averaging just 0.60 sprays for *B. tabaci* control and 1.46 sprays for all arthropod pests during the last 3 years.^124^

**Figure 22:**
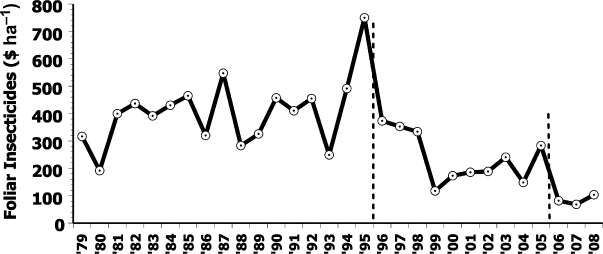
Statewide average foliar insecticide costs including application costs (in $ US per hectare) invested by growers to control all arthropod pests, adjusted for inflation, 1979–2008, Arizona, USA (derived from^124^). IGRs, transgenic Bt cotton, and the Arizona IPM plan for cotton was introduced in 1996. Pink bollworm eradication activities began in 2006 and flonicamid for Lygus control became available in 2006–2007.

IC was critical to the activation of this remarkable trend, which represents a reduction of ca 70% in foliar insecticide use compared with pre-IGR deployment, and enabled significant economic and environmental savings as well. The authors estimate cumulative post-deployment savings in control costs and insect-related yield loss prevention of over $ 201 599 000 in the last 14 years (derived from data in reference 124). Furthermore, in spite of a general trend of more expensive per application costs, growers invested a 30 year record low in insecticides in 2007 (<$69 ha^−1^ versus record high 1995 levels of >$749 ha^−1^) (Fig. 22). The insecticide load on the environment creates untold savings to non-target organisms and in risks to human health. After a decade's high usage of insecticides in 1995, there has been about a 0.77 million kg reduction in insecticides used annually, starting in 2006.

The detailed synthesis here provides for a rare documented case of IC, where the interplay between biological and chemical control is explicitly measured and exploited for augmented pest control. With the added and coincident significant benefits of Bt cotton (1996–) and later eradication efforts (2006–) for the pink bollworm, and more selective alternatives to acephate for *L. hesperus* control, growers are now able better to exploit and build upon IC of *B. tabaci*. This effort has engendered a culture of respect for the in-field mortality dynamics and cross-commodity interactions that govern the *B. tabaci* system, and has led to large IPM subscription rates by the agricultural community in Arizona. Broad-spectrum chemistry still plays a role, but is relegated to the option of last resort and generally deferred to the final seasonal spray, if needed at all. The result has been an unprecedented stability of ecosystem services and major economic and environmental gains in Arizona cotton that has extended to benefit the entire agroecosystem of the region.
